# Equity in adherence to antiretroviral therapy among economically vulnerable adolescents living with HIV in Uganda

**DOI:** 10.1080/09540121.2016.1176681

**Published:** 2016-07-08

**Authors:** Laura Gauer Bermudez, Larissa Jennings, Fred M. Ssewamala, Proscovia Nabunya, Claude Mellins, Mary McKay

**Affiliations:** ^a^ Columbia University School of Social Work, International Center for Child Health and Asset Development, New York, NY, USA; ^b^ Department of International Health, Social and Behavioral Interventions Program, Johns Hopkins Bloomberg School of Public Health, Baltimore, MD, USA; ^c^ School of Social Service Administration, University of Chicago, Chicago, IL, USA; ^d^ HIV Center for Clinical and Behavioral Studies, New York State Psychiatric Institute and Columbia University Medical Center, New York, NY, USA; ^e^ McSilver Institute for Poverty Policy and Research, Silver School of Social Work, New York University, New York, NY, USA

**Keywords:** Equity, HIV, youth, Uganda, ART adherence, assets, Suubi

## Abstract

Studies from sub-Saharan Africa indicate that children made vulnerable by poverty have been disproportionately affected by HIV with many exposed via mother-to-child transmission. For youth living with HIV, adherence to life-saving treatment regimens are likely to be affected by the complex set of economic and social circumstances that challenge their families and also exacerbate health problems. Using baseline data from the National Institute of Child and Human Development (NICHD) funded Suubi+Adherence study, we examined the extent to which individual and composite measures of equity predict self-reported adherence among Ugandan adolescents aged 10–16 (*n* = 702) living with HIV. Results showed that greater asset ownership, specifically familial possession of seven or more tangible assets, was associated with greater odds of self-reported adherence (OR 1.69, 95% CI: 1.00–2.85). Our analyses also indicated that distance to the nearest health clinic impacts youth’s adherence to an ARV regimen. Youth who reported living nearest to a clinic were significantly more likely to report optimal adherence (OR 1.49, 95% CI: 0.92–2.40). Moreover, applying the composite equity scores, we found that adolescents with greater economic advantage in ownership of household assets, financial savings, and caregiver employment had higher odds of adherence by a factor of 1.70 (95% CI: 1.07–2.70). These findings suggest that interventions addressing economic and social inequities may be beneficial to increase antiretroviral therapy (ART) uptake among economically vulnerable youth, especially in sub-Saharan Africa. This is one of the first studies to address the question of equity in adherence to ART among economically vulnerable youth with HIV.

## Introduction

The success of antiretroviral therapy (ART) for HIV depends greatly on an individual’s ability to access antiretroviral (ARV) medicines and strictly adhere to the required drug regimen (Peltzer & Pengpid, 2013). Yet, inequality in who has access to life-saving ARV medicines as well as the economic and social supports needed to remain in treatment, can contribute to non-adherence (Baltussen et al., 2013). Given the resource constraints in sub-Saharan Africa, examining variations in access to ART by population group has been a long-standing priority of HIV equity research (Baltussen et al., 2013; Johnson, 2012; Tromp, Michels, Mikkelsen, Hontelez, & Baltussen, 2014). Health equity can be defined as “the absence of unfair and avoidable or remediable differences in health among population groups defined socially, economically, demographically, or geographically” (International Society for Equity in Health, 2005). In contrast, health inequities are considered present when the consequences of those same characteristics result in adverse health outcomes (Braveman & Gruskin, 2003; Fylkesnes et al., 2013). For example, studies have shown that ARV drugs are less accessible for men and children as compared to women (Baltussen et al., 2013; Johnson, 2012; Tromp et al., 2014). Equity in access to ART has also been examined along clinical practices of treating patients on a first-come basis as compared to treating those who are most ill or likely to benefit from treatment (Baltussen et al., 2013; Cleary, Mooney, & McIntyre, 2010; Kalanda, Makwiza, & Kemp, 2007; Mendelsohn, Spiegel, Schilperoord, Cornier, & Ross, 2014). Less commonly, HIV equity concerns have also grappled with ARV allocation on the basis of treatment adherence (Kimmel, Daniels, Betancourt, Wood, & Prosser, 2012) and the integration of men into home-based HIV/AIDS care (Newman, Fogarty, Makoae, & Reavely, 2011).

Despite the growing literature on equity in HIV care and treatment, little has been studied regarding equity in adherence to ART. Studies have shown that utilization of ARV medications is disproportionally low among the poor (Cleary et al., 2011; Tromp et al., 2014; Tsai, Chopra, Pronyk, & Martinson, 2009). Such low utilization has been attributable in part to the “inverse equity hypothesis” which posits that individuals of higher socio-economic status are the first to benefit from new health initiatives, in this case, ARV drug and adherence support services (Cleary et al., 2011; Hargreaves, Davey, & White, 2013; Victora, Vaughan, Barros, Silva, & Tomasi, 2000). In addition to barriers in accessing and initiating ART, the poor face barriers related to continuing or adhering to drug regimens. Commonly cited reasons for non-adherence reflect both economic and social concerns, such as lack of finances to purchase ARV medicines (Gusdal et al., 2009; Ramadhani et al., 2007), transportation to clinic appointments (Emenyonu, Thirumurthy, & Muyindike, 2010; Mukherjee, Ivers, Leandre, Farmer, & Behforouz, 2006; Tuller et al., 2010), food insecurity (Hardon et al., 2007; Weiser et al., 2010), and lack of social support (Peltzer, Friend-du Preez, Ramlagan, & Anderson, 2010; Wouters, Van Damme, Van Loon, van Rensburg, & Meulemans, 2009). As a result, we have focused the term “equity” to represent absence of differences in medication adherence by both economic and social factors. Assessing how ARV non-adherence is distributed can indicate not only whether HIV adherence interventions are sufficiently pro-poor, but also whether segments of the relatively poor or socially marginalized are able to equally adhere to and benefit from ARV services.

To begin to respond to this question, this study assesses if missing ARV doses among adolescents with HIV is significantly associated with economic and social inequities, as measured by individual- and community-level indicators. Examining economic and social inequities in ARV treatment interruptions is an increasingly recognized research priority (Cleary et al., 2011; Cooke, Tanser, Bärnighausen, & Newell, 2010; Hargreaves et al., 2013; Kalanda et al., 2007). Nevertheless, only a few studies have examined such disparities in ARV adherence (Cleary et al., 2011; Cooke et al., 2010; Cornell, Myer, Kaplan, Bekker, & Wood, 2009; Govindasamy et al., 2011; Orrell, Bangsberg, Badri, & Wood, 2003; Tsai et al., 2009), and most have been limited to traditional demographic (i.e., age and gender) and equity measures (i.e., wealth quintiles and concentration indices) that overlook broader socio-economic domains and largely exclude youth as a sample population (Chakraborty, Firestone, & Bellows, 2013; Tromp et al., 2014). To date, the literature on adherence among adolescents has given nearly exclusive focus to biomedical and psychosocial factors (Bhana et al., 2014; Naar-King et al., 2006; Williams et al., 2006). This paper examines whether differential adherence outcomes are associated with specific economic and social attributes among Ugandan adolescents living with HIV. Based on the findings, we discuss implications for addressing adherence disparities among poor and disadvantaged youth in low-resource settings.

## Methods

### Study design

This paper used baseline data from the SUUBI+Adherence Project, a five-year longitudinal randomized control trial examining the effect of a family-based financial asset intervention on ARV adherence for youth living with HIV in Uganda. Inclusion criteria for youth included: (1) having tested positive for HIV (confirmed by medical report and aware of status); (2) living within a family; (3) being 10–16 years of age; (4) having been prescribed ART; and (5) being enrolled in care at a participating medical clinic. Method of HIV acquisition, perinatal, or behavioral was not differentiated for the purposes of this study. However, given the mean age of respondents and incidence of mother-to-child transmission in the region, it can be plausibly inferred that the majority of adolescents participating in the study acquired HIV through perinatal infection.

### Setting

Over 190,000 children (ages 0–17) are living with HIV in Uganda (UNICEF, 2015), with the 9.8% prevalence rate in the study region of greater Masaka, higher than the national average of 7.3% (Government of Uganda, 2013). ARV roll-out began in Uganda in 2004. Nonetheless, with 40% of Ugandans living on less than $1.25 USD per day (World Bank, 2015), those at economic disadvantage often find associated costs of treatment prohibitive, negatively affecting their access and adherence to medication (Emenyonu et al., 2010; Tuller et al., 2010; Weiser et al., 2010).

### Measures

The measures used were adapted from previous studies in the region (Ismayilova, Ssewamala, & Karimli, 2012; Ssewamala et al., 2010; Ssewamala & Ismayilova, 2009), and included questions developed specifically for HIV and AIDS affected youth as well as pre-established and culturally relevant assessment measures.

The primary outcome of analysis for this paper is self-reported ARV adherence. Each respondent was asked to recount the number of days they had missed at least one dose of ARV medication in the last month. This variable was dichotomized with those indicating no doses were missed as adherent (code = 1) and those who had missed one or more doses as non-adherent (code = 0).

Three control variables were included in the model – age in years, dichotomized at the sample mean, (code = 0 if 10–12 years, code = 1 if 13–16 years), gender (code = 0 if male, code = 1 if female), and number of HIV medications (code = 0 if one medication, code = 1 if two or three medications).

Eight economic equity variables were assessed and subdivided into two composites:

*Assets & Employment:* (1) asset ownership, equally weighted summation of tangible household assets – such as livestock, gardens, modes of transportation, and land – measured dichotomously as low possession (6 or fewer reported assets, code = 0) or high possession (7 or more reported assets, code = 1); (2) caregiver employment in the formal labor market (no = 0, yes = 1); (3) available cash savings (no = 0, yes = 1); (4) parent or caregiver participating in a formal banking institution (no = 0, yes = 1); and (5) material housing value, measured as low-value (mud or hut, code = 0) or high value (brick, code = 1).

*Food Security*: (6) number of meals per day, reported as low consumption (1 or fewer, code = 0) or high consumption (2 or more, code = 1); (7) frequency of eating meat or fish in the prior week (1 or fewer, code = 0) or (2 or more, code = 1); and (8) breakfast consumption on day of interview (no = 0, yes = 1).

Six social equity variables were examined that represented the respondent’s access and proximity to community resources and availability of social support: (1) enrolled in school at baseline (no = 0, yes = 1); (2, 3, 4) distance to the school, water source, and health clinic, each with the same categorization (code = 0 if “far”, “very far”, “no clinic”, or “don’t know”; code = 1 if “near” or “very near”); (5) electricity in the home (no = 0, yes = 1); and (6) social support for medication adherence (no = 0, yes = 1).

Composite scores for the equity measures were created by quantifying the number of responses coded “1” under each themed dichotomous predictor variable. Dummy variables were then created for each composite with those marked as “0” within the lowest quintile of summed scores.

### Analysis

Data were analyzed using SPSS Version 22 (IBM Corp., Armonk, NY, USA). Bivariate comparisons of self-reported ARV adherence and measures of economic and social equity were examined using binary logistic regression, controlling for age, gender, and number of HIV medications. Following the test of individual predictors, we examined composite measures of economic and social equity to understand the summative effects of inequity. Each composite was sequentially added through multivariate logistic regression to understand whether its inclusion improved the overall model fit. All analyses were considered significant at *p* < .05.

## Results

### Sample demographic characteristics

Screening procedures were carried out on 990 adolescents, excluding from the study those who failed to meet inclusion criteria, predominately, lack of awareness of HIV status, under or over age, or attendance at a health clinic outside of the study’s catchment area, resulting in a final analytical sample of 702 respondents (Table 1). Of the 702 respondents, 52% (*n*  =  365) were between the ages of 10 and 12 and 48% (*n*  =  337) were between 13 and 16 years of age. Girls represented 56.4% (*n* =  396) of the sample. Just under one quarter of respondents indicated taking one medication (22.8%, *n*  =  160) compared with 77.1% taking two to three medications (541).

**Table 1.** Demographic characteristics of enrolled adolescents, aged 10–17, at baseline.

Mean age in years (±SD)	12.4 (2.0)
Younger adolescents (10–12 years) (%, *n*)	365 (52.0)
Older adolescents (13–16 years) (%, *n*)	337 (48.0)
Proportion of girls (*n*, %)	396 (56.4)
Number of reported ARV medications prescribed	
At least 1 ARV medication	160 (22.8)
Two to three ARV medications	541 (77.1)
Missing	1 (0.1)

### Distribution of economic and social equity measures

Respondents whose family had six or fewer household assets represented 9.7% of the sample (*n*  =  68) with those listing seven or more assets at 90.3% (*n*  =  634) (Table 2). Only 10.7% of respondents had caregivers in the formal labor market (75). Individual savings was reported by 29.2% of the sample (*n*  =  205), while knowledge of a parent or caregiver having an account at a bank or Savings and Credit Cooperative (SAACO) was indicated by 28.9% (*n*  =  203). Living in a mud or hut house was reported by 12.7% of respondents (*n*  =  89) while 87.3% indicated living in a brick home with or without a cement floor (*n*  =  613).

**Table 2.** Bivariate distribution of economic and social equity variables in adolescents at baseline (*N * =  702).

	*n* (%)
Economic equity variables	
Assets & savings	
Asset ownership	
Low possession (six or fewer reported assets)	68 (9.7)
High possession (seven or more reported assets)	634 (90.3)
Employment of adolescent’s caregiver in formal labor market	
No	627 (89.3)
Yes	75 (10.7)
Available cash savings	
No	497 (70.8)
Yes	205 (29.2)
Parent or caregiver participation in formal banking institution	
Does not have a banking account (or does not know if parent/caregiver has account)	499 (71.1)
Has a banking account	203 (28.9)
Material housing value	
Mud or hut only (low-value)	89 (12.7)
Brick built (higher-value)	613 (87.3)
Composite Assets, Employment, & Savings	
Mean number of economic equity measures reported (± SD) [Out of 5 above items]	2.46 (1.0)
Proportion of adolescents reporting all five (*n* = 5) equity measures	19 (2.7)
Proportion of adolescents reporting no (*n* = 0) equity measures	13 (1.9)
Food security	
Number of meals per day	
Low consumption (1 or fewer)	85 (12.1)
High consumption (2 or more)	616 (87.7)
Missing	1 (0.1)
Frequency of eating meat or fish in past week	
Low consumption (1 or fewer)	364 (51.9)
High consumption (2 or more)	338 (48.1)
Breakfast on day of interview	
Low consumption (did not eat)	139 (19.8)
High consumption (did eat)	563 (80.2)
Composite food security	
Mean number of economic equity measures reported (± SD) [Out of three above items]	2.2 (0.8)
Proportion of adolescents reporting all three (*n* = 3) equity measures	282 (40.2)
Proportion of adolescents reporting no (*n* = 0) equity measures	30 (4.3)
Social equity variables	
Primary or secondary school enrollment	
No	89 (12.7)
Yes	613 (87.3)
Physical and social proximity to school	
Far or very far (over 3 kilometers)	91 (13.0)
Near or very near (approx. 0–3 kilometers)	611 (87.0)
Physical and social proximity to water source	
Far or very far (over 3 kilometers)	12 (1.7)
Near or very near (approx. 0–3 kilometers)	690 (98.3)
Physical and social proximity to health clinic	
Far or very far (over 3 kilometers) or don’t know	158 (22.5)
Near or very near (approx. 0–3 kilometers)	542 (77.2)
Missing	2 (0.3)
Electricity in home	
No	538 (76.6)
Yes	164 (23.4)
Social support for ARV adherence	
No	98 (14.0)
Yes	604 (86.0)
Composite social equity	
Mean number of social equity measures reported (± SD) [Out of six above items]	4.6 (0.9)
Proportion of adolescents reporting all six (*n* = 6) economic equity measures	80 (11.4)
Proportion of adolescents reporting no (*n* = 0) economic equity measures	0 (0.0)

Twelve percent of respondents (*n*  =  85) reported having one or fewer meals per day, while the majority (87.7%, *n*  =  616) reported having at least two meals per day. Respondents who consumed meat or fish one or fewer times in the past week represented 51.9% of the sample population (*n* =  364) compared with those who ate meat or fish two or more times (48.1%, *n*  =  338). Nearly one-fifth of the respondents (19.8%, *n*  =  139) reported not eating breakfast the day of the interview.

Most respondents were enrolled in school (87%, *n*  =  613) with 13% of respondents (*n*  =  91) indicating their school as “far or very far” and 87% (*n*  =  611) reporting the school as “near or very near” (Table 2). Nearly all respondents reported having a water source that they considered to be “near or very near” (98.3%, *n* =  690); while 77.2% (*n*  =  542) reported the same for the health clinic. Less than one quarter of participants reported having electricity in their home (23.4%, *n*  =  164). Fourteen percent (*n*  =  98) responded that no one assisted them with their medication while 86% (*n*  =  604) indicated some form of social support for adherence.

### Equity in ARV adherence by economic factors

Seventy-one percent (70.6%, *n*  =  494) of respondents reported optimal adherence to ART, compared to 29.4% (*n*  =  206) who reported having missed medication one or more times in the last 30 days. In bivariate analyses, higher asset possession was significantly associated with optimal ARV adherence by a factor of 1.69 (OR 1.69, 95% CI: 1.00–2.85) (Table 3). Greater odds of adherence were also associated with cash savings (OR 1.07, 95% CI: 0.75–1.54); parent or caregiver participation in formal banking institution (OR 1.16, 95% CI: 0.80–1.67); higher material housing value (OR 1.27, 95% CI: 0.79–2.05); greater frequency of meals (OR 1.49, 95% CI: 0.92–2.40); more frequent intake of meat or fish in the previous week (OR 1.05, 95% CI: 0.75–1.45); and consumption of breakfast the day of the interview (OR 1.10, 95% CI: 0.73–1.65), though these findings were not statistically significant. Bivariate analyses found no observable relationship between reported adherence and parent or caregiver employment in the formal sector (OR 0.61, 95% CI: 0.37–1.01). In multivariate analysis, controlling for age, gender, and number of HIV medications, higher scores on the *Assets & Employment* composite measure were significantly associated with adherence (aOR 1.70, 95% CI: 1.07–2.70) (Table 4). We found no relationship between adherence and the *Food Security* composite measure.

**Table 3.** Binary logistic regression – Individual measures associated with adherence.

	OR	OR 95% CI	*P*-Value
Economic equity variables			
Asset and employment composite	1.66*	1.06–2.61	.026
Asset ownership	1.69*	1.00–2.85	.049
Employment of adolescent’s caregiver in formal labor market	0.61	0.37–1.01	.055
Available cash savings	1.27	0.79–2.05	.321
Parent or caregiver participation in formal banking institution	1.07	0.75–1.54	.701
Material housing value	1.16	0.80–1.67	.434
Food security composite	1.00	0.66–1.51	.991
Number of meals per day	1.49	0.92–2.40	.102
Frequency of eating meat or fish in past week	1.05	0.75–1.45	.796
Breakfast on day of interview	1.10	0.73–1.65	.642
Social equity variables			
Social equity composite	1.41	0.87–2.28	.168
Primary or secondary school enrollment	0.85	0.57–1.59	.952
Proximity to school	1.13	0.70–1.82	.625
Proximity to water source	1.66	0.51–5.34	.399
Proximity to health clinic	1.49*	1.02–2.18	.040
Electricity in home	1.00	0.68–1.48	.993
Social support for ARV adherence	1.14	0.72–1.81	.590

*Significance at *p *< .05 noted in bold.

**Table 4.** Sequential multivariable logistic regression – composite measures associated with adherence.

Adjusted odds ratio (aOR)	Model 1 aOR (95% CI)	Model 2 aOR (95% CI)	Model 3 aOR (95% CI)	Model 4 aOR (95% CI)
Demographic factor				
Age	0.92 (0.66–1.28)	0.92 (0.66–1.28)	0.91 (0.66–1.27)	0.93 (0.67–1.30)
Gender	1.73* (1.25–2.40)	1.73* (1.25–2.41)	1.75* (1.26–2.44)	1.73* (1.24–2.41)
Number of HIV medications	1.19 (0.81–1.74)	1.18 (0.80–1.74)	1.18 (0.80–1.73)	1.19 (0.81–1.75)
*Economic equity*				
Assets & employment	–	1.70* (1.09–2.67)	1.74* (1.10–2.76)	1.70* (1.07–2.70)
Food security	–	–	0.90 (0.59–1.38)	0.88 (0.57–1.35)
*Social equity*	–	–	–	1.37 (0.84–2.23)
Model comparison parameters				
*X*^2^	–	16.94, df = 4	17.17, df = 5	18.68, df = 6
*P* value	–	.002	.004	.005

*Significance at *p *< .05 noted in bold.

### Equity in ARV adherence by social factors

Respondents living near to a health clinic had greater odds of optimal adherence (OR 1.49, 95% CI: 1.02–2.18) (Table 3). Proximity to school (OR 1.13, 95% CI: 0.70–1.82); distance to water source (OR 1.66, 95% CI: 0.51–5.34) and social support for adherence (OR 1.14, 95% CI: 0.72–1.81) were associated with greater odds of adherence, though not statistically significant. No difference in adherence odds was observed for electricity within the home or enrollment in school. In multivariate analysis, the composite measure for social support was associated with increased odds of adherence (OR  =  1.37, 95% CI: 0.84–2.23), though the findings did not meet the threshold for statistical significance (Table 4).

## Discussion

Appeals for equity in healthcare delivery are not a new phenomenon (Marmot, Friel, Bell, Houweling, & Taylor, 2008; Sen, 2002; Victora et al., 2003). Yet, balancing principles of health equity with feasibility, particularly in low-resource settings, is a distinct challenge with present efforts to address inequities in HIV treatment falling short (Baltussen et al., 2013). This study examined the association between several economic and social equity variables on differential adherence to life-saving ARV medications among adolescents living with HIV. Whereas prior research has focused on the psychosocial and biomedical determinants of adherence among youth (Bhana et al., 2014; Naar-King et al., 2006; Williams et al., 2006), to the best of our knowledge, this study is among the first to examine equity in ARV adherence among adolescents in sub-Saharan Africa by economic and social disadvantage.

Our findings indicate that distance to the health clinic and number of household assets were both associated with higher self-reported adherence among Ugandan youth. Previous research supports the association between health clinic distance and adherence (Cooke et al., 2010), with transportation cost a primary concern (Emenyonu et al., 2010; Mukherjee et al., 2006; Tuller et al., 2010). Differing from the limited research that exists on household assets and adherence, formerly demonstrating no association (Cooke et al., 2010), our findings suggest familial wealth resources do have a significant and positive impact on medication adherence among Ugandan youth living with HIV. The additional observed association between the composite asset and employment measure with adherence to ART suggests there may be a cumulative effect to economic disadvantage with youth who routinely fall at the lower end of the spectrum on measures of economic stability less likely to adhere.

The study found no significant association between food security and ARV adherence. One possible explanation for the lack of association is that economic constraints have a greater impact on obtaining transportation to attend HIV clinical appointments or fill ARV prescriptions. On the other hand, while distance to a health clinic was independently found to be a factor in ARV adherence, a significant association between the composite social equity measure and adherence was not observed. It is plausible that our measures of social equity (i.e., school enrollment, proximity to resources, and social support for medication adherence) were not fully illustrative of adolescents’ relative advantage or disadvantage in social contexts. For example, social stigma in school settings may have an inverse effect on ARV medication adherence, with prior research in the United States suggesting fear of status disclosure and social ostracism reasons for non-adherence (Rao, Kekwaletswe, Hosek, Martinez, & Rodriguez, 2007; Rintamaki, Davis, Skripkauskas, Bennett, & Wolf, 2006). More research is needed in the context of sub-Saharan Africa to understand whether differential adherence among adolescents is impacted by varying levels of social support within schools, among peers, or within communities. These dimensions of social equity were unmeasured in this analysis but may be relevant for future study or intervention.

In addition, our analysis identified gender as a significant variable associated with adherence. Girls were more likely to report optimal compliance with their ARV regimen. This contradicts the existing literature that being female is also positively associated with non-adherence (Berg et al., 2004; Puskas et al., 2011; Tapp et al., 2011). As current inquiry on gender disparities is largely limited to adult populations, there is a need to better understand and address gender inequities in ARV adherence among adolescents in low-resource settings. Do odds of adherence change as young girls marry and bear familial responsibilities? What are the specific challenges to adherence for adolescent boys? Would information on ART be more effective for boys if disseminated at sporting events, food markets, or rites of passage? Such data would inform interventions seeking to sustain ARV adherence from adolescence to adulthood.

While this study provides insight on factors associated with adherence among adolescents living with HIV, the findings also have practice implications, namely that economic and social determinants of adherence be given due priority in the design and development of programs affecting youth with HIV in sub-Saharan Africa. Interventions that aim to improve financial assets, enable participation in formal financial institutions; and provide geographically closer HIV treatment services such as through mobile clinics may offer promising returns for greater equity in ARV uptake and adherence among poor adolescent populations.

Future research on inequities in ARV utilization would benefit greatly from a core set of common measures. Echoing prior calls for such standardization (Chakraborty et al., 2013; Tromp et al., 2014), the formation of economic and social equity indicators for application in public health research is suggested. Such measures may also be applied as a multi-dimensional screening tool for youth prescribed ART, assessing resource gaps across economic and social gradients and predicting potential adherence interruptions. The routine monitoring of inequities will benefit global health practitioners and social marketers in the strategic design of programs to reach those most vulnerable to non-adherence. Further, a consistent flow of data that enumerates inequity can serve to improve advocacy efforts and increase accountability at local, national, and international levels.

## Limitations

Our findings were limited by the use of self-reported measures of adherence which are prone to overestimation. Nevertheless, of the available measures, self-report has been found to be practical, low-cost, significantly associated with viral load, and sufficiently reliable to draw conclusions on ARV uptake (Kabore et al., 2014; Simoni et al., 2006; Usitalo, Leister, & Tassiopoulos, 2013).

Review of the findings should also consider that the analysis was limited to available lines of inquiry on food security, potentially affecting interpretation. Designed to measure broader poverty status, the SUUBI+Adherence questionnaire was not intended to extensively evaluate food security at the household level. Thus, future studies aiming to examine the effect of economic and social equity on health outcomes, may wish to consider inclusion of a validated scale at trial outset.

Lastly, this study employed only quantitative assessments of equity. Further research is needed to understand qualitatively how adolescents interpret their economic advantage or disadvantage and how that influences their ability to maintain ARV adherence.

## Conclusion

When seeking to improve uptake of ART in low-resource settings, even where medication is provided without cost, particular attention must be paid to effectively reach children and youth at greatest economic and social disadvantage. Our findings suggest that mutually reinforcing resource constraints may negatively affect medication adherence in this population, potentially resulting in worse health outcomes for youth living with HIV.
